# Dynamic modeling of the growth of spoilage fungi on sweet cherries under fluctuating temperatures

**DOI:** 10.3389/fmicb.2026.1858080

**Published:** 2026-06-23

**Authors:** Shaohua Xing, Wenming Xing, Xinguang Fan, Xiangquan Zeng

**Affiliations:** 1 School of Food Engineering, Ludong University, Yantai, China; 2 Department of Food Science, Purdue University, West Lafayette, IN, United States

**Keywords:** fluctuation temperature, predictive model, spoilage, spoilage fungi, sweet cherry

## Abstract

This study aimed to develop a predictive growth model for spoilage fungi on sweet cherries under fluctuating temperatures to improve shelf life management of sweet cherries in the supply chain. Spoilage fungi were inoculated onto sterile fresh sweet cherries, which were then stored at a range of temperatures (5, 10, 15, 20, 25, and 30 °C) to monitor fungal growth. The Baranyi model was used as the primary model to predict the growth of spoilage fungi. A square-root model and a linear regression equation were used as secondary models to describe the temperature dependence of the maximum specific growth rate (*μ_max_*) and lag phase duration (*λ*), respectively. The Baranyi model effectively characterized the growth of spoilage fungi, as validated by an excellent fit to the data (all *R^2^* > 0.900) and low prediction errors (root mean square error *[RMSE]* close to zero). The high *R^2^* values (0.927 and 0.974) and low *RMSE* values (0154 and 0.143) for the secondary models indicated accurate estimation of the primary model parameters. Based on the integration of primary and secondary models, an integrated dynamic model was developed for prediction. Validation at fluctuating temperatures proved that the predictive model exhibited good stability and reliability. This model can accurately predict the growth of spoilage fungi, thereby forecasting the shelf life of sweet cherries and guiding their freshness maintenance.

## 1 Introduction

Sweet cherry (*Prunus avium L.*) is abundant in nutrients, bright, and unique in flavor (Aubert et al., 2024; Gu et al., 2022). It is suitable for direct fresh consumption and can be further processed into a variety of commercial products (Sumic et al., 2013; Xing et al., 2025a). Previous research has revealed that sweet cherries contain abundant bioactive compounds, particularly polyphenols, which confer significant health-promoting properties, including antioxidant, hypoglycemic, hypolipidemic, and anticancer effects (Domínguez-Rodríguez et al., 2021; Kazazic et al., 2024; Wani et al., 2014). China has become the world’s largest producer and consumer of sweet cherries, leading in both cultivation area and import volume (Chockchaisawasdee et al., 2016). Nevertheless, sweet cherries feature a thin peel, soft texture, and abundant juice. It is prone to mechanical injury and subsequent microbial contamination (Chen et al., 2025).

The surface of sweet cherries has many spoilage microorganisms. Once the peel is damaged, microorganisms will quickly invade and multiply, resulting in fruit decay (Zhang et al., 2021). Relevant investigations have shown that postharvest decay caused by microbial infection accounts for over half of total sweet cherry postharvest losses in China (Gu et al., 2022). The main microorganisms causing postharvest decay of sweet cherry fruit are fungi, including *Penicillium, Monilinia, Rhizopus, Mucor, Alternaria,* and other genera (Feliziani et al., 2013; Morca et al., 2022; López et al., 2016).

Temperature is a key factor affecting the growth of spoilage fungi in sweet cherries (Zhang et al., 2021). Toivonen (2014) studied the effects of different hydrocooling temperatures on the quality of sweet cherries, finding that lower hydrocooling temperatures slowed the growth of microorganisms and improved the quality of sweet cherries. Zhang et al. (2021) demonstrated a positive correlation between ambient temperature and the abundance of spoilage fungi in the microbial communities inhabiting the surface of sweet cherries. Therefore, low-temperature conditions are usually required for the storage and transportation of sweet cherries (Wang et al., 2023). However, temperature fluctuations frequently occur in the logistics chain because of improper handling and outdated equipment (Ndraha et al., 2018). Temperature abuse promotes the proliferation of spoilage fungi (Yang et al., 2024). The rapid proliferation of spoilage fungi not only induces severe fruit decay but also produces mycotoxins that pose potential risks to human health (Ahmad et al., 2024). Hence, it is essential to investigate how spoilage fungi change with temperature on sweet cherries for accurately predicting the shelf life of sweet cherries.

Microbial growth kinetics is an established and reliable method used to predict microbial changes in various vegetables and fruits and to assess their shelf lives (Zhao et al., 2022). Tsironi et al. (2017) developed a microbial growth model to predict the shelf life of ready-to-eat fresh-cut salads during the cold-chain supply. Seyed and Reza (2022) used the Baranyi model to determine the growth of microorganisms and modeled the shelf life of pomegranate juice under various storage temperatures. However, the growth dynamics of spoilage fungi and their impact on the shelf life of sweet cherries after harvest remain insufficiently investigated.

Therefore, the objective of this research was to develop a practical model for estimating and predicting the growth of spoilage fungi on sweet cherries under fluctuating temperatures. This study will provide technical support for management decisions by understanding the changes in spoilage fungi on sweet cherries during the supply chain.

## 2 Materials and methods

### 2.1 Materials

Sweet cherries (*Prunus avium* L. cv. ‘Mei Zao’) were harvested at commercial maturity (90% ripe) with an average single fruit weight of 10 ± 2 g. Fruits were sourced from an orchard in Yantai, China, and transported to the laboratory within 1 h. Fruits were selected for uniform size and color and were free from mechanical injury, insect infestation, and pathogen infection. Before the experiment, the samples were soaked in chlorine dioxide solution (30 ppm) for 3 min, rinsed with sterile water, and then air-dried in a cool, well-ventilated environment.

### 2.2 Spore suspension preparation

Spore suspensions were obtained from five strains of spoilage fungi. Among them, *Penicillium expansum* (BNCC 122814, blue mold: soft rot of the pulp, with blue–green powdery mold on the surface), *Monilinia fructicola* (BNCC 113562, brown rot: fruit turns brown and rots, producing grayish-brown mold, eventually shriveling into a mummy), *Rhizopus stolonifer* (BNCC 357852, soft rot: fruit becomes extremely soft and exudes juice, covered with white cottony mycelium and black granules), and *Mucor racemosus* (BNCC 195604, mucor rot: fruit softens and collapses) were obtained from the China Microbial Culture Preservation Center (CMCPC, Beijing, China). *Alternaria alternata* (LD-101, black spot rot: black, sunken lesions appear on the fruit surface) was obtained from Ludong University’s Fruit and Vegetable Preservation Laboratory. The revived culture was inoculated onto the potato dextrose agar (PDA) (HB0233-12, Haibo Biotechnology Co., Ltd., Qingdao, China) surface to obtain isolated colonies. Once adequate sporulation was observed, 10–15 mL of sterile 0.85% NaCl saline was added to the plate surface, and the spore mass was gently scraped using a sterile cell scraper. Then, the resulting suspension was filtered through multiple layers of sterile gauze into a new sterile tube (Hengchao Laboratory Equipment Co., Ltd., Nantong, China). The filtered spore suspension was mixed with a vortex mixer (XH-C, Jierian Instrument & Equipment Co., Ltd., Wuxi, China) for 1–2 min. The concentration of the spore suspension was determined by hemocytometer enumeration. Finally, the five strains were mixed in equal proportions to form a standardized spore inoculum.

### 2.3 Inoculation

The sterilized sweet cherries were carefully placed into sterile sampling bags (Kangyue Medical Packaging Co., Ltd., Anqing, China). Each bag contained approximately 25 g of fruit. The cherry samples were inoculated with 1 mL of a mixed fungal spore suspension to obtain an initial level of approximately 2.0 log CFU/g, followed by 2 min of shaking to ensure even surface coverage. Finally, the samples were stored at temperatures of 5, 10, 15, 20, 25, and 30 °C. The number of fungal colonies on sweet cherries was determined at an interval of 4.5 days at 5 °C, 3 days at 10 °C, 2 days at 15 °C, 1 day at 20 °C and 25 °C, and 0.5 days at 30 °C, respectively.

### 2.4 Spoilage fungi enumeration

The counting of spoilage fungi was carried out according to the GB 4789.15–2016 (National Health and Family Planning Commission, 2016). Sweet cherries in the bag were transferred aseptically into 225 mL of sterile diluent and homogenized with a tapping homogenizer (PC-PJ-400, Puchun Measure Instrument Co., Ltd., Shanghai, China) for 1–2 min. The resulting homogenate was subjected to 10-fold serial dilutions. Subsequently, 1 mL of the appropriate dilution was pipetted onto Rose Bengal medium and incubated in an incubator at 28 ± 1 °C for 4–5 d to record the number of colonies.

### 2.5 Modeling

#### 2.5.1 Primary model

The growth kinetics of spoilage fungi in sweet cherries were characterized by the Baranyi model (Baranyi and Roberts, 1994), as shown in Equation 1. The number of microbial counts is expressed as the common logarithm of colony-forming units (CFU) per gram of sample (log CFU/g). Since the prediction results of the Baranyi model are usually in the form of natural logarithms (ln CFU/g), unit conversion is required during modeling.


$$y_{\left(t\right)}=y_{0}+\mu_{\max}A-\ln(1+\frac{e^{\mu_{\max}A}-1}{e^{y_{\max}-y_{0}}})$$ (1)

Where $A=t+\frac{1}{\mu_{\max}}\ln[e^{-\mu_{\max}t}+e^{-\mu_{\max}\lambda }-e^{-\mu_{\max}(t+\lambda )}]$

Where *y_0_* and *y_max_* are the initial and final cell counts (ln CFU/g), *λ* is the lag phase duration (h), *t* is the time (h), and *μ_max_* is the maximum specific growth rate (h^−1^). All parameters were estimated by non-linear fitting in Origin 2021 (OriginLab Corporation, MA, United States).

#### 2.5.2 Secondary model

Researchers often model the effect of temperature on microbial growth using a secondary model (Xing et al., 2025b). A square-root model (Equation 2) was proposed by Ratkowsky et al. (1982) to quantify the temperature dependence of the maximum specific growth rate.


$$\sqrt{\mu_{\max}}=b(T-T_{\min})$$ (2)

Where *T* is the temperature (°C), *T_min_* is the lowest temperature for microorganisms’ growth, and *b* is a regression coefficient. By fitting the value of *μ_max_*, the parameter *b* and the minimum growth temperature *T_min_* of spoilage fungi were obtained.

The relationship between *μ_max_* and *λ* was modeled using a linear regression equation (Equation 3) (Goh et al., 2025). The equation was as follows:


$$\ln\lambda =a_{1}-b_{1}\times \ln\mu_{\max}$$ (3)

Where a_1_ and b_1_ are regression coefficients, and λ is the lag phase duration (h).

#### 2.5.3 Dynamic models

Baranyi and Roberts (1994) developed a mathematical model (Equation 4) that was coupled with the secondary models in Equations 2, 3 to analyze the dynamic growth behavior of spoilage fungi under fluctuating temperatures.


$$\left\{ \begin{array}{c} \frac{\mathrm{dy}}{\mathrm{dt}}=\frac{1}{1+e^{-Q(t)}}\mu_{\max}T(t)[1-e^{\left(y_{\left(t\right)}-y_{\max}\right)}]\\ \frac{\mathrm{dQ}}{\mathrm{dt}}=\mu_{\max}T(t)\\ q_{0}=\frac{1}{e^{h_{0}}-1} \end{array}\right.$$ (4)

The initial conditions were set as *y(0)* = *y₀* and *Q(0)* = *ln(q₀)*, with *q₀* derived from the *h₀* value. *h_0_* is a parameter that characterizes the physiological state of the microorganisms, which equals *μ_max_* × *λ*. For numerical integration, the fourth-order Runge–Kutta method was implemented and run in the MATLAB environment.

#### 2.5.4 Goodness-of-fit statistics

The accuracy of the models was assessed using the coefficient of determination (*R^2^*) (Xing et al., 2025b). An *R^2^* value closer to 1 denotes better fitting performance. Meanwhile, model precision was assessed using the root mean square error (*RMSE*), with lower values reflecting higher accuracy (Chai and Draxler, 2014). The formulas for calculating *R^2^* and *RMSE* are presented in Equations 5, 6, respectively.


$$R^{2}=1-\frac{\sum_{i=1}^{n}{\left(\overset{\wedge }{y_{i}}-y_{i}\right)}^{2}}{\sum_{i=1}^{n}{\left(y_{i}-\bar{y}\right)}^{2}}$$ (5)

$$\text{RMSE}=\sqrt{\frac{\sum_{i=1}^{n}{\left(y_{i}-\overset{\wedge }{y_{i}}\right)}^{2}}{n}}$$ (6)

Where *n* is the sample size, *y_i_* and *ŷ_i_* are the i-th observed and predicted values, respectively, and *ȳ* is the mean of observations. Goodness-of-fit, expressed as *R^2^* and *RMSE*, was evaluated using Microsoft Excel 2020.

### 2.6 Model verification

Referring to China Federation of Logistics and Purchasing (CFLP) (2021), the dynamic model was validated under two distinct temperature profiles: slowly changing profile: 28 °C for 12 h, 12 °C for 10 h, 20 °C for 20 h, 25 °C for 10 h, and 6 °C for 8 h, which was repeated for 9 cycles, and rapidly changing profile: 28 °C for 4 h, 10 °C for 3 h, 22 °C for 1 h, 6 °C for 13 h, 18 °C for 1 h, 10 °C for 3 h, 20 °C for 1 h, and 6 °C for 14 h, which was repeated for 15 cycles. Temperature fluctuations were simulated using a programmable temperature test chamber (KHTH80-40, Kunhai Testing Equipment Co., Ltd., Huizhou, China). The Baranyi model was applied in conjunction with the experimental temperature data and secondary models of *μ_max_* and *λ* to dynamically predict fungal growth.

To validate the dynamic prediction model, the acceptable prediction zone (APZ) method was used (Oscar, 2005). Prediction error (PE) was then calculated using Equation 7.


$$\mathrm{PE}=y_{o}-y_{p}$$ (7)

Where y_o_ and y_p_ are the experimental observations and model estimates, respectively. Following the criteria established by Moon et al. (2018) and Wang et al. (2022), model predictions falling within the APZ range of −1.0 to 0.5 log CFU/g were regarded as reliable.

### 2.7 Statistical analysis

All measurements were performed in triplicate. Origin 2021 software (OriginLab Corp., Northampton, MA, United States), MATLAB R2022 (MathWorks, Inc., Massachusetts, United States), and Microsoft Excel 2020 (Microsoft Corporation, Inc., United States) were used for statistical analysis of the experimental data to establish the model.

## 3 Results and discussion

### 3.1 Growth of spoilage fungi and fitting of the primary model on sweet cherry under different temperatures

**Figure 1:**
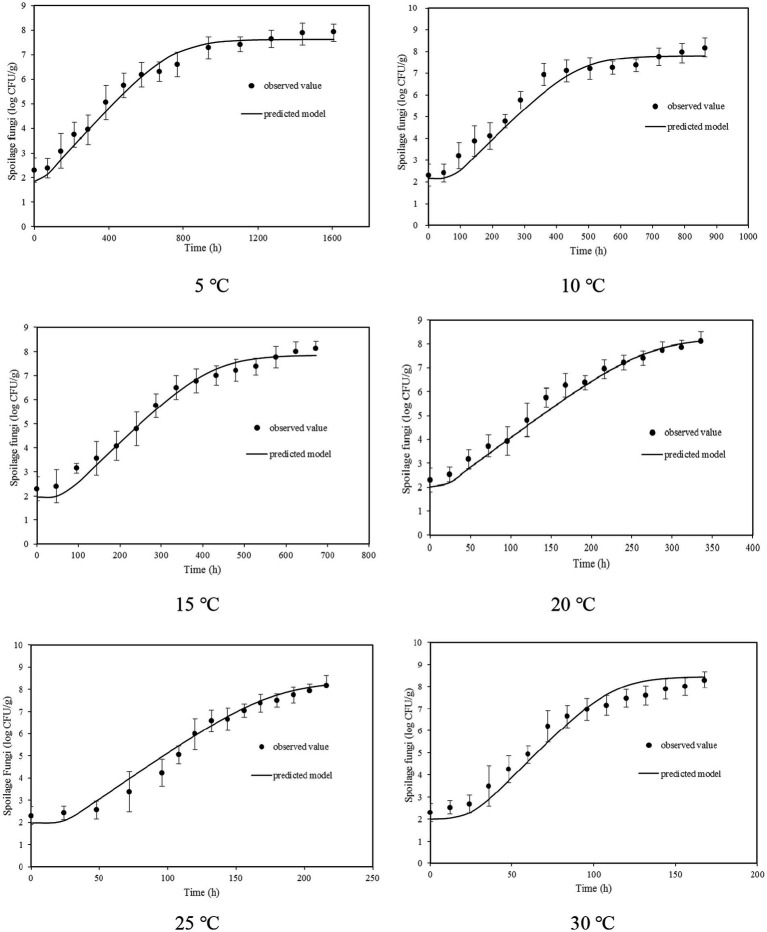
Observed the growth of spoilage fungi on sweet cherries at different temperatures.

Fungi are the main spoilage microorganisms that cause cherry rot (Chu et al., 2025; Saito et al., 2016). As shown in Figure 1, the growth of spoilage fungi on sweet cherries showed typical sigmoidal patterns at different storage temperatures. The initial number of spoilage fungi was approximately 1.992 ± 0.125 log CFU/g in the lag phase (Table 1), which was consistent with a previous study stating that the initial surface fungal count of freshly harvested sweet cherries was approximately 2.25 log CFU/g (Zhang, 2020). The lag time *λ* decreased with increasing temperature, ranging from 100.833 h to 7.249 h (Table 1). The *μ_max_* of the spoilage fungi increased with storage temperature, from 0.009 ± 0.001 h^−1^ to 0.076 ± 0.052 h^−1^ in the exponential growth phase (Table 1). These values were higher than those reported in previous studies by Pei et al. (2022), who reported that the *μ_max_* of *Fusarium graminearum* was 0.020 ± 0.003 h^−1^ at 25 °C on wheat. This discrepancy was mainly attributed to differences in fungal species and food substrates, both of which can affect the maximum specific growth rate (Gil-Serna et al., 2015). In the stationary phase, the maximum number of spoilage fungi reached approximately 8.067 ± 0.024 log CFU/g with storage time. This finding was consistent with a previous study, which reported that the number of molds in corn at stationary phase was 7.84 log CFU/g (Yue et al., 2017). The Baranyi model was used for non-linear fitting of the growth trend of spoilage fungi on sweet cherries at 5–30 °C. Table 1 displays the fit of the primary model obtained at different temperatures. The *R^2^* value of the fitted model ranged from 0.955 to 0.976, and the *RMSE* ranged from 0.290 to 0.464, indicating that the primary model accurately described the growth dynamics of spoilage fungi on sweet cherries.

**Table 1:** Fitting parameters of spoilage fungi growth on sweet cherries at different temperatures.

Temperature (°C)	*y_max_* (log CFU/g)	*μ_max_* (1/h)	*y_0_* (log CFU/g)	*λ*(h)	*R^2^*	*RMSE*
5	7.623 ± 0.414	0.009 ± 0.001	2.276 ± 0.156	100.833	0.976	0.302
10	7.801 ± 0.224	0.015 ± 0.002	2.158 ± 0.314	81.652	0.955	0.441
15	7.854 ± 0.346	0.018 ± 0.016	2.170 ± 0.254	60.257	0.970	0.338
20	8.301 ± 0.132	0.027 ± 0.028	2.011 ± 0.175	28.855	0.978	0.290
25	8.372 ± 0.221	0.042 ± 0.001	2.374 ± 0.058	16.833	0.974	0.334
30	8.452 ± 0.118	0.076 ± 0.052	2.262 ± 0.435	7.249	0.957	0.464

y_0_, initial growth quantity of spoilage fungi (log CFU/g, converted from ln CFU/g); y_max_, maximum growth quantity of spoilage fungi (log CFU/g, converted from ln CFU/g).

### 3.2 Secondary models of spoilage fungi

**Figure 2:**
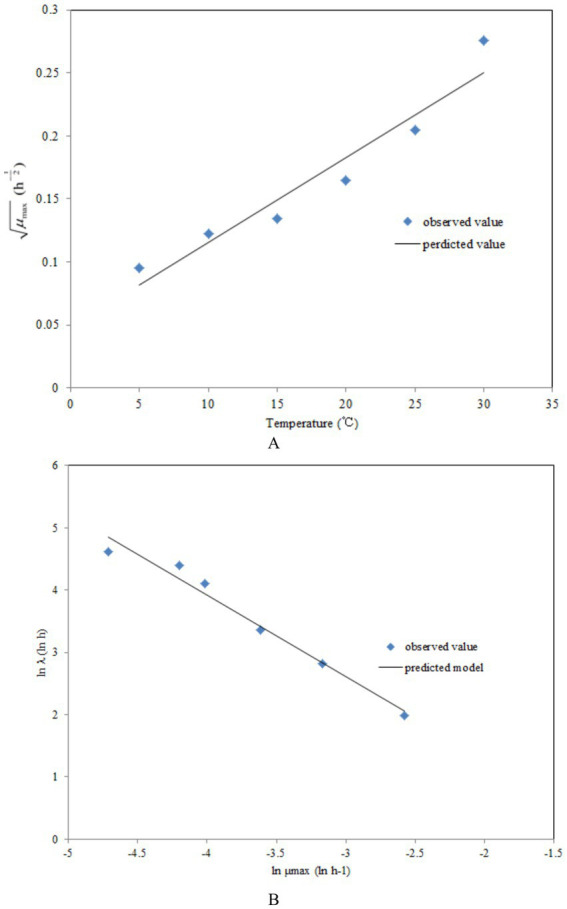
Secondary models of temperature effect on the maximum specific growth rate μmax **(A)** and lag phase duration *λ* **(B)** of spoilage fungi on sweet cherries.

A square-root model well described the maximum specific growth rate (*μ_max_*) of spoilage fungi on sweet cherries within the temperature range of 5–30 °C. As shown in Figure 2A, a strong linear correlation was identified between temperature and the square root of *μ_max_* (*R^2^* = 0.927, *RMSE* = 0.154). The lowest growth temperature of the spoilage fungi was −7.044 °C. Previous studies had reported similar outcomes. Sardella et al. (2018) modeled the growth of pear postharvest fungal isolates and determined that the minimum temperature of *Penicillium expansum* growth was −8.78 °C. The corresponding fitting equation is presented as Equation 8.


$$\sqrt{\mu_{\max}}=0.0684\times [T-(-7.044)]$$ (8)

A negative linear dependence of ln *λ* on ln *μ_max_* was observed, as shown in Figure 2B. The estimated coefficient *a_1_* was −1.336, and the *b_1_* coefficient was 1.315. The linear function (Equation 9) demonstrated an excellent fit to the data, with a high *R^2^* of 0.974 and a low *RMSE* of 0.143.


$$\ln\lambda =-1.336-1.315\times \ln\mu_{\max}$$ (9)

## 4 Model validation

**Figure 3:**
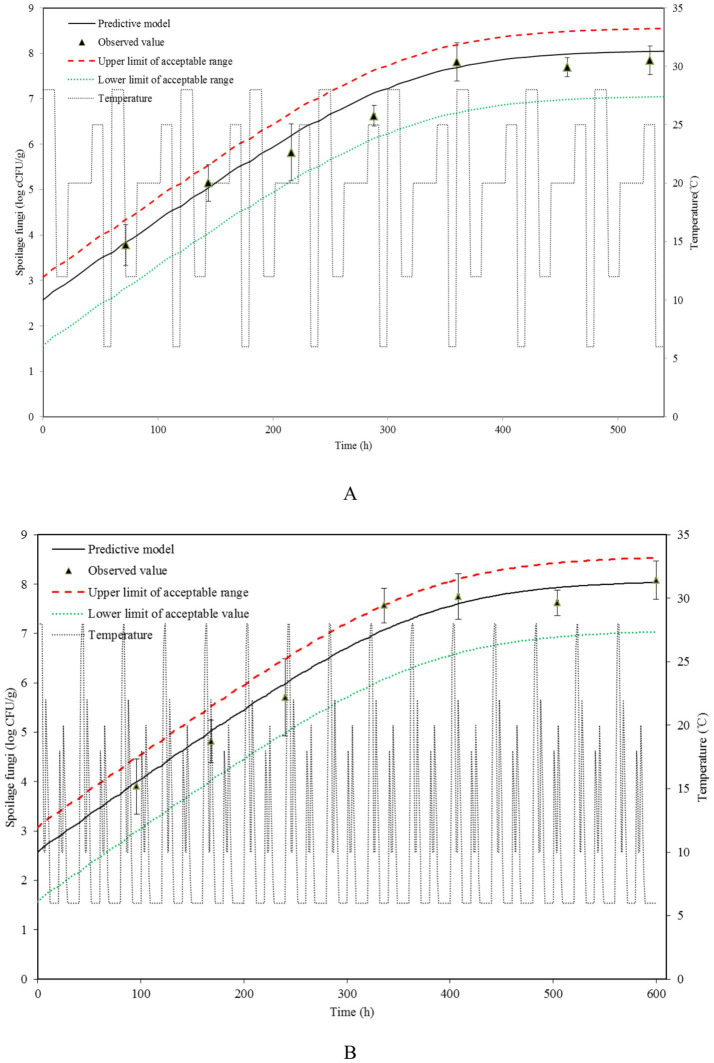
Dynamic model of spoilage fungi growth on sweet cherries at different fluctuating temperature profiles. **(A)** slowly changing profile. **(B)** rapidly changing profile.

Growth data collected both under a slowly changing profile and a rapidly changing profile validated the dynamic model. Figure 3 shows that temperature fluctuations significantly altered the slope of the predicted growth curves, while a strong correlation remained between predicted and observed values. This confirmed the model’s strong predictive power for temperature abuse, demonstrating that isothermal kinetic parameters provide a reliable basis for dynamic prediction.

**Figure 4:**
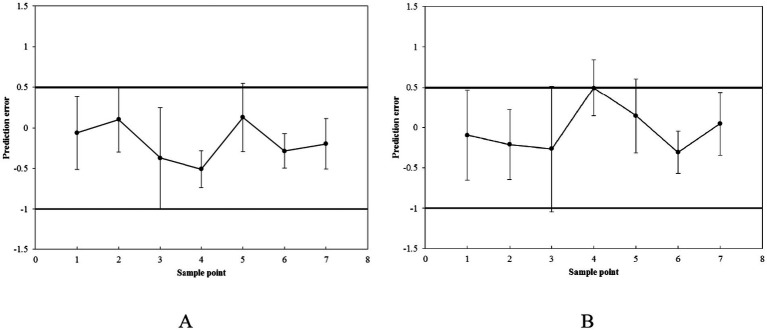
APZ for spoilage fungi growth at seven sample points under different fluctuating temperature profiles. **(A)** slowly changing profile; **(B)** rapidly changing profile.

Researchers frequently use the acceptable prediction zone (APZ) approach to assess the predictive performance of microbial growth models of various foods (Goh et al., 2025). The prediction error (*PE*) values of two temperature fluctuation profiles are illustrated in Figure 4. Nearly all detected data points of spoilage fungi in sweet cherries fell within the APZ, ranging from −1.0 to 0.5 log CFU/g. This further confirmed that the dynamic prediction model constructed using isothermal kinetic parameters achieved satisfactory agreement with experimental data under variable temperature conditions.

## 5 Conclusion

This study established growth prediction models targeting a mixed spoilage fungal consortium naturally occurring on sweet cherry. In this study, the growth of spoilage fungi in sweet cherries was measured at different temperatures (5 °C, 10 °C, 15 °C, 20 °C, 25 °C, and 30 °C). The growth kinetic model of spoilage fungi was constructed using the Baranyi model. All coefficients of determination (*R^2^*) were greater than 0.900, indicating that the Baranyi primary model can describe the growth of spoilage fungi well (all *R*^2^ > 0.900). By integrating the primary model with secondary models (the square-root model and linear regression equation) that describe the temperature dependence of key kinetic parameters, a dynamic predictive model was successfully established. The dynamic model can predict the growth of spoilage fungi in sweet cherries under fluctuating temperatures and effectively monitor the freshness and shelf life of sweet cherries within a 5–30 °C fluctuating temperature range.

However, sweet cherries used in this study were inoculated with five types of spoilage fungi, which differed from those naturally present on commercially harvested sweet cherries in terms of species composition and relative abundance. Future studies should further study the practicality of the model in predicting the spoilage fungi in naturally harvested sweet cherries. In addition, the temperature fluctuation ranges adopted in the model were empirically set in advance, which may fail to fully reflect actual environmental conditions. Subsequent studies should use temperature profiles obtained from real cold-chain monitoring data to conduct further verification.

## Data Availability

The original contributions presented in the study are included in the article/supplementary material, further inquiries can be directed to the corresponding author.
